# Mega Cisterna Magna: Current Perspectives and Future Directions—A Literature Review

**DOI:** 10.1155/rrp/9591364

**Published:** 2025-11-23

**Authors:** Masoud Pishjoo, Daniel Kheradmand, Mohammad Safdari, Zohre Safdari, Mojtaba Mashhadinejad

**Affiliations:** ^1^Department of Neurosurgery, Shohada Hospital, Qaen, South Khorasan, Iran; ^2^Department of Neurosurgery, Mashhad University of Medical Sciences, Mashhad, Iran; ^3^Department of Neurosurgery, Zahedan University of Medical Sciences, Zahedan, Iran; ^4^Department of Radiology, Shohada Hospital, Qaen, South Khorasan, Iran

**Keywords:** cerebellar malformations, differential diagnosis, mega cisterna magna, neuroimaging, posterior fossa

## Abstract

The posterior fossa anomaly known as mega cisterna magna (MCM) is defined as an enlarged cisterna magna that is more than 10 mm in the anteroposterior dimension, without concomitant vermian or cerebellar malformations. Although this condition is usually discovered incidentally during prenatal or neuroimaging examinations, its clinical significance and long-term effects are still being studied and discussed. The prognosis and treatment of MCM depend on its differentiation from other posterior fossa malformations, including Blake's pouch cyst, Dandy–Walker variant, and arachnoid cysts. In addition to exploring debates surrounding MCM and potential clinical applications, this review attempts to summarize the state of knowledge currently available on the anatomical, embryological, clinical, and radiological aspects.

## 1. Introduction

### 1.1. Mega Cisterna Magna (MCM) Definition

A cisterna magna enlargement of more than 10 mm in the anteroposterior dimension on midsagittal imaging is called MCM [1]. When the ventricular and cerebellar structures are intact, it is regarded as a benign anatomical variant rather than a true malformation [2]. It is frequently discovered incidentally during fetal ultrasound or magnetic resonance imaging (MRI), especially when other conditions are being routinely screened for or evaluated [2, 3]. The absence of vermian hypoplasia, mass effect, and communication with the fourth ventricle are the main characteristics that set MCM apart from other abnormalities of the posterior fossa [4].

### 1.2. A Synopsis of the History

The development of MRI in the 1980s and 1990s, which made it possible to visualize the posterior fossa structures in greater detail, led to a greater understanding of MCM as a unique neurodevelopmental abnormality [1, 3]. MCM was once thought to be a benign anatomical variation. However, with the development of neuroimaging techniques, especially fetal and pediatric MRI, MCM is now regarded as a mild alteration in the group of posterior fossa abnormalities, the others being more severe [5].

### 1.3. Significance of Identifying MCM From Other Anomalies

In the past, these fluid-filled spaces were confused with arachnoid cysts or Dandy–Walker malformations (DWMs) since the resolution of old CT and early US imaging was not good enough [5]. Differentiation from other conditions is critical due to different prognostic and treatment implications [6, 7]. For example, while surgery is required for DWM, MCM is typically asymptomatic and requires no treatment [4]. Misinterpretation of imaging findings can lead to anxiety for parents, unnecessary follow-up imaging, and inappropriate neurosurgical consultation, particularly in prenatal clinics.

The clinical significance of MCM is still a matter of controversy among researchers. Although most individuals are asymptomatic, recent research indicates potential associations with neurodevelopmental retardation, psychiatric disease, or brain structural syndromes are all an emerging hypothesis [8–10]. Proper diagnosis, classification, and counseling are thus important, particularly in prenatal cases.

### 1.4. Objective and Scope of the Investigation

The present review has the goal of presenting an extensive review of MCM, encompassing its anatomical features, embryologic origin, epidemiology, clinical presentation, imaging features, differential diagnosis, and management. We also pinpoint contemporary controversies and suggest potential research avenues to unveil the importance of this condition.

## 2. Anatomy and Embryology of the Cisterna Magna

### 2.1. Normal Anatomy and Development of the Posterior Fossa

**Figure 1:**
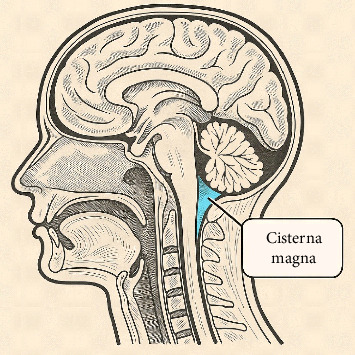
Anatomy of cisterna magna.

The most intricate and deepest cranial fossa is the posterior cranial fossa, which contains the brainstem and cerebellum. It is delimited anteriorly by the clivus and the dorsum sellae, posteriorly by the occipital bone, and laterally by the temporal and parietal bones [11]. Cisterna magna, located between the cerebellum and the dorsal side of the medulla oblongata, is the largest subarachnoid cistern and is also known as the cerebellomedullary cistern (Figure 1). Its enlargement or malformation is considered a developmental anomaly, and this structure is vital for CSF circulation [1]. This structure communicates with the fourth ventricle through the median aperture (foramen of Magendie) and laterally with the pontine cistern via the lateral apertures (foramina of Luschka). In addition, its enlargement or malformation may signal concealed developmental disorders [12].

### 2.2. Embryological Development of the Cisterna Magna

The development of the fossa posterior begins around the fifth or sixth week of embryonic development, where the hindbrain (rhombencephalon) starts to form both the brainstem and the cerebellum. While the fourth-week prenatal scanning shows the neural tube starting the process of dividing into primary brain vesicles, in the growing mid-stage of pregnancy, around the fourth week, the hindbrain (rhombencephalon) is further divided into metencephalon and myelencephalon [13]. The ventral sections of both the metencephalon (rhombic lips) differentiate into the dorsal parts and then grow toward the midline region, where they can develop dorsal and medial regions of the cerebellum [14].

Cisterna magna develops as a subarachnoidal formation gap situated between the medulla and the pons and is of significant relevance to the construction due to the openings of the fourth ventricular apertures. Foramen of Magendi is a large opening in the subarachnoid space that normally occurs between the 10th and 12th week of gestation, which allows cerebrospinal fluid (CSF) to increase adequate flow into the spinal cord subarachnoidal space and gives rise to its expansion [13, 15]. Failure in this activity can result in multiple anomalies of the posterior fossa.  Table 1 summarizes key embryological milestones in the development of the cisterna magna. MCM is hypothesized to arise from delayed patency of the foramen of Magendie (Weeks 10–12) or altered CSF dynamics, without primary cerebellar maldevelopment [13, 15].

### 2.3. How MCM Fits Into the Spectrum of Posterior Fossa Anomalies

Within this broader spectrum of cystic malformations of the posterior fossa lies MCM, with other examples being the DWM, Blake's pouch cyst, and arachnoid cysts. Key differentiating features of posterior fossa anomalies are summarized in Table 2, adapted from established diagnostic criteria [5, 11, 15]. MCM differs from the DWM in that there is no hypoplasia of the vermis, and rotation of the cerebellum and the posterior fossa is vertical, whereas cystic dilation of the fourth ventricle is present alongside an enlarged posterior fossa, and there is cystic dilation of the fourth ventricle. DWM has features such as cystic dilation of the fourth ventricle. It has been suggested that MCM arises due to ineffective absorption of CSF or delayed foramen of Magendie opening, not due to primary cerebellar maldevelopment [16].

MCM has also been hypothesized to result from delayed or incomplete expansion of the vermis or from abnormal CSF dynamics leading to excessive accumulation in the cisternal space without affecting cerebellar architecture [17]. The boundary between normal anatomical variation and pathological enlargement remains blurred. This ambiguity leads to the question of whether MCM should be considered a variant of normal or mild malformation [10, 18].

## 3. Epidemiology of the Cisterna Magna

### 3.1. Incidence and Prevalence

The true incidence of MCM remains hard to establish due to its asymptomatic nature and incidental diagnosis in neuroimaging studies. Estimates from fetal MRI and postnatal imaging indicate that MCM is present in about 0.4%–1.5% of normal prenatal ultrasound examinations; this incidence might be less if rigorous diagnostic criteria are applied [1, 5].

In research focused on population-based prenatal assessments, isolated MCM occurs in approximately 1 in every 1000 to 5000 pregnancies [2, 3]. A study carried out by Pishjoo et al. in 2025 in eastern Iran indicated that the prevalence of this condition was assessed at 9.1%. Furthermore, it was found that the prevalence rate was considerably elevated in males in comparison to females. Importantly, instances of significantly enlarged MCM were documented solely among male individuals [19].

### 3.2. Clinical Characteristics

Isolated MCM is often asymptomatic and found incidentally, though it can infrequently be associated with developmental delay or other neurological findings [15]. During follow-up, the size of the cisterna magna may remain stable or even decrease as development continues, especially if the case has been diagnosed prenatally [20].

### 3.3. Association With Other Anomalies or Syndromes

While MCM is often seen as an isolated finding, it can also be associated with a number of anomalies. Structural anomalies of the fetus, such as agenesis of the corpus callosum, cerebellar hypoplasia, and Dandy–Walker continuum disorders, have been documented in as high as 20%–40% of cases of nonisolated MCM [16, 21]. Chromosomal anomalies such as Trisomy 18 and Trisomy 13 have also been reported to be associated with an enlarged cisterna magna. These fetal evaluations and possible chromosomal abnormalities underscore the need for careful fetal evaluation and genetic counseling when an enlarged cisterna magna is found on ultrasound [22, 23].

There have been studies on possible associations with 16p11.2 duplication syndrome, neurodevelopmental delay, and very rare mixed associations with renal or craniofacial anomalies; however, the association must not be confused with causation [8, 17, 18]. Although there could be possible associations, MCM diagnosed as truly isolated, with no other associated sonographic or clinical findings, has a good prognosis [9].

## 4. Etiology and Pathophysiology

The exact etiology of MCM has yet to be completely defined, and multiple developmental and physiological processes have been proposed to be factors in its development. MCM has been regarded as a benign anatomical variant, but there is emerging evidence to suggest that it may represent a more subtle type of malformation in the posterior fossa [1].

It is postulated that the etiology of MCM is multifactorial, with several hypotheses put forth as regard its cause, including developmental arrest of the posterior membranous area, alterations in CSF dynamics, or secondary expansion from outside of the cerebellum [15, 24].

### 4.1. Developmental Arrest Hypothesis

The leading hypothesis involves the explanation of MCM as a result of a developmental arrest of the inferior vermis during embryonic formation of the posterior fossa [25]. The incomplete or delayed fusion of the vermian hemispheres may allow a cisternal space to be disrupted enough that the cistern remains expanded, despite the absence of frank vermian hypoplasia. However, cerebellar morphology is preserved in MCM, so the developmental disruption is assumed to be mild [5].

### 4.2. CSF Flow

Another explanation involves abnormal CSF flow. MCM may develop because of excess CSF entering the cisterna magna due to transient or localized constrictions in the fetal CSF pathways. There are specific sites of potential obstruction, such as the foramina of Luschka and Magendie [2, 6]. These functional blockages could expand the cisternal area without causing usual ventriculomegaly or compressing surrounding structures.

### 4.3. Association With Other Posterior Fossa Anomalies

MCM, many researchers believe, exists in some continuum of posterior fossa anomalies such as Blake's pouch cyst, arachnoid cysts, and Dandy–Walker variants (DWVs) [26]. It is important to note that MCM has a normal vermian rotation, an intact cerebellar hemisphere, and a normal tentorium as an isolated anomaly; therefore, it is structural and functional [3].

### 4.4. Genetic and Acquired Factors

While MCM is most commonly isolated, some studies have suggested possible genetic associations, including 16p11.2 microduplication, which may impact abnormal hindbrain development or CSF regulation [18]. In rare cases, there are reports of MCM as well as renal, ear, or craniofacial anomalies, which may suggest common embryological pathways or syndromic associations [17].

Environmental factors or intrauterine insults have not been consistently implicated in MCM formation, and an acquired cause has not been identified in the literature.

## 5. Clinical Presentation

### 5.1. Prenatal and Neonatal

In the prenatal setting, it is most often detected on second-trimester ultrasound as a widened or enlarged cisterna magna having an anteroposterior dimension greater than 10 mm, with a normal-appearing vermis and fourth ventricle not altered [3]. In the absence of anomalies, isolated MCM has an excellent prognosis and developmentally fits a similar neurodevelopmental trajectory as infants without MCM [6].

### 5.2. Pediatric

MCM may be identified in infants and children when imaging is conducted for unrelated concerns, such as developmental delay, macrocephaly, or seizures. However, studies indicate that isolated MCM has no reliable neurological signs [7]. There have been rare reports of MCM seen along with mild hypotonia, developmental delay, or behavioral disorders, but there is no reliable evidence that these are caused by MCM [8].

### 5.3. Adult

In adults, more commonly, diagnosis is incidental in brain MRIs or CT scans done for other reasons (e.g., headache, dizziness, and trauma) [10]. There are occasional reports of neuropsychiatric symptoms (e.g., mood disorder and psychotic or cognitive symptoms) being related to MCM; however, these cases are also uncommon, with most studies confounded by comorbidities [17, 18].

## 6. Neuroimaging Features

Accurate neuroimaging is needed to make a diagnosis of MCM and differentiate it from other posterior fossa malformations. The main imaging tools used in the clinical setting are ultrasound (i.e., prenatally) and MRI (i.e., pre- and postnatally), while CT is occasionally used in older children or adult cases.

### 6.1. MRI and CT Findings

The radiographic definition of MCM is an enlarged cisterna magna defined as > 10 mm in the anteroposterior diameter taken from the posterior aspect of the vermis to the inner table of the occipital bone [5]. The typical findings of MCM are as follows:

• Enlarged retrocerebellar CSF space
• Normal morphology and size of the vermis
• No upward elevation of the tentorium
• Normal fourth ventricle
• Developed and normally appearing cerebellar hemispheres [2, 3]

**Figure 2:**
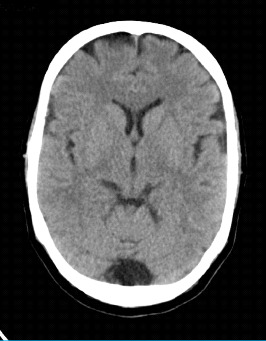
MCM in CT scan.

**Figure 3:**
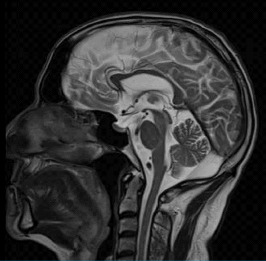
MCM in MRI.

On CT, MCM may be seen as a well-defined low density in the posterior fossa (Figure 2), but CT does not provide soft tissue detail to evaluate the cerebellar vermis and tentorium precisely. MCM is seen on T2-weighted MRI as a CSF-intensity signal posterior to the cerebellum, which is in continuity with the fourth ventricle and does not have a mass effect (Figure 3) [1].

### 6.2. Prenatal Imaging Criteria

MCM is typically identified prenatally on second-trimester ultrasound as an enlarged cisterna magna > 10 mm in size. The diagnosis is confirmed with fetal MRI, and more importantly, it allows for a better assessment of the vermis and tentorium and other cerebellar anatomy that is needed to exclude more complex anomalies [6].

### 6.3. Differentiation From Other Posterior Fossa Cystic Lesions

Differentiating MCM from other posterior fossa cystic anomalies is important due to the significant differences in the prognosis, as well as clinical management. The most important differential diagnoses are the following.

1. Arachnoid Cyst:
  • Appearance is a noncommunicating CSF collection
  • May exert mass effect on adjacent structures
  • Can cause displacement of the cerebellum and fourth ventricle [11]
2. Blake's Pouch Cyst:
  • Failure of the regression of Blake's pouch
  • Communicates with the fourth ventricle but often elevates the vermis and compresses the cerebellum [26]
3. DWM:
  • Defined as a vermis hypoplasia/agenesis, cystic dilation of the fourth ventricle, and elevation of the tentorium
  • Significant cerebellar and midline abnormalities [11]
4. DWV:
  • Partial vermian hypoplasia with mild fourth ventricle enlargement
  • Considered less severe than classic DWM, but most cases have reported neurodevelopmental delay [25]
5. Vermian Hypoplasia Without Cyst:
  • May have a normal-sized cisterna magna
  • Vermis minimally appears small, dysplastic, or rotated, which may be visualized on a mid-sagittal plane fetal MRI [27]

## 7. Clinical Implications and Management

The clinical importance of MCM primarily hinges on whether it occurs in isolation or alongside other neurological or systemic issues. MCM is regarded as a pathological condition only if it is linked with additional structural, genetic, or neurodevelopmental abnormalities. In the absence of these factors, it is typically considered a benign anatomical variant. Many studies in the existing literature have identified MCM as an incidental and benign finding, particularly in prenatal evaluations. While the majority of MCM cases are indeed benign, it is important to note that a small fraction—approximately less than 5%—may be associated with other anomalies that could necessitate medical intervention. Understanding the context in which MCM occurs is crucial for determining its clinical significance and guiding appropriate management [4].

### 7.1. Management of Incidental MCM

For the majority of cases, MCM is discovered incidentally on MRI imaging for another unrelated reason, especially in the antenatal setting, ultrasound, or MRI when being assessed for developmental delay or trauma [1]. Assuming MCM is an isolated finding and that imaging reveals a normal cerebellar vermis, normal tentorium, and normal fourth ventricle, there should not be any intervention. The only routine follow-up after postnatal diagnosis of incidental findings to rule out other anomalies that might have been detected normally would be occasionally performed.

• Clinical neurological exams
• Developmental monitoring during infancy and early childhood
• Neuroimaging would only be performed if deemed clinically warranted, if symptoms arise, or if MCM is found with other anomalies [6]

Studies have shown that isolated MCM previously reported in the literature did not have a deleterious impact on neurodevelopment, especially in the absence of other anomalies [2].

### 7.2. Clinical Decision-Making for MCM

Table 3 summarizes evidence-based management strategies for MCM. Isolated cases require no intervention, while associated anomalies or symptoms warrant targeted evaluation [5, 6, 20].

### 7.3. Follow-Up Strategies

Postnatal imaging will be recommended in the following scenarios.

• When prenatal imaging is suboptimal
• When ultrasound and MRI findings are discordant
• When associated anomalies or ventriculomegaly are suspected
• When there is a change in the size of the cisterna magna or any symptoms in the postnatal period [3].

Neurological and developmental follow-up of these children is recommended on a periodic basis, even though mostly asymptomatic in nature.

### 7.4. Neurosurgical Indications

Surgical management of MCM is exceptionally rare, and beyond being indicated for isolated findings, even when surgery is necessary, there may be limited treatment options for morbidity. These patients may require a neurosurgical consultation for the following:

• Hydrocephalus from an obstructive process
• Significant mass effect based on a presumed misdiagnosis (such as a large arachnoid cyst mistaken for MCM)
• Symptomatic patients, especially those presenting with headaches, imbalance, or cranial nerve signs/deficits [5].

In such cases, it is necessary to re-evaluate imaging to confirm the diagnosis and assess for other possible causes, such as arachnoid cysts or Blake's pouch cysts, which may require fenestration or shunt [5]. In the rare instance where a true, isolated MCM is believed to be the direct cause of obstructive hydrocephalus or significant mass effect, surgical intervention—typically involving endoscopic third ventriculostomy or, less commonly, cystoperitoneal shunting—may be considered [5].

### 7.5. Prognostic Implications

The prognosis of isolated MCM is excellent. Most infants and children remain neurologically normal. However, the prognosis would be less favorable if the MCM is associated with the following:

• Chromosomal abnormalities
• Other CNS anomalies
• Neurodevelopmental syndromes [5]

## 8. Controversies and Current Debates

One of the significant current disputes is whether MCM should be regarded only as a variant or when it exists with a spectrum of mild malformation. It has been suggested that in people with MCM, subtle cerebellar or cognitive dysfunctions may be present and not recognized [28].

There is also considerable ambivalence around the long-term neurodevelopmental effects of MCM in children. Some reports suggest no adverse effects, while others report minor effects on motor or language development, but there is a lack of consistency across findings and reports on this issue [29, 30].

Terminology and classification remain issues for debate concerning MCM that require further clarification, particularly when trying to differentiate MCM from Blake's pouch cyst and vermian hypoplasia, which may be directly related to imaging appearance but are not developmentally related [15, 31].

## 9. Future Directions and Research Gaps

Prospective studies are needed to better characterize the natural history of MCM and the true prevalence of the condition in the population at large. The identification of imaging biomarkers that predict clinical outcomes or can help in differentiating MCM from pathologic cysts in the early stages is important.

In addition, the genetics studies would clarify whether there are any underlying molecular reasons for isolated MCM, especially with familial clustering or syndromic considerations. In addition, standardized imaging protocols and terminology to achieve a consistent diagnosis and management across centers would benefit further studies on MCM.

## 10. Conclusion

MCM is still a difficult diagnostic and clinical entity due to its overlap with other posterior fossa anomalies and incidental findings. Advancements in fetal and neonatal neuroimaging have greatly enhanced recognition of MCM and separation from other posterior fossa anomalies (e.g., Blake's pouch cyst, arachnoid cyst, and DWVs). However, ongoing inconsistencies in terminology and limitations to the precision of prenatal diagnostics continue to be troublesome for clinicians and radiologists.

In nearly all cases, especially when isolated, it does not have a detrimental neurodevelopmental outcome, nor will it require a surgical intervention. There are rare symptomatic phenotypes of MCM, and the association with other anomalies warrants evaluation to confirm the role of assessment, regular follow-up, and, in selected cases, genetic evaluation.

It is essential to be multidisciplinary in the clinical approach to MCM, especially during prenatal settings when counseling depends on merging imaging findings with possible genetic implications and family concerns. In rare cases, MCM needs surgery, but being aware of the differential diagnosis of MCM is important to avoid unnecessary operative procedures and facilitate appropriate referral and follow-up.

In conclusion, as we move forward, the standardization of classification systems, longitudinal outcome trajectories, and genetic correlations will be critical in demystifying the latent level of presentation and complications involved with MCM. It is through these processes that neurosurgeons, pediatricians, and radiologists can gain a better understanding of this complex neurodevelopmental condition and improve patient management.

## Figures and Tables

**Table 1:** Embryological timeline of the cisterna magna.

Gestational week	Developmental event	Clinical relevance to MCM
Weeks 4-5	Hindbrain (rhombencephalon) forms	Early disruptions may affect the posterior fossa CSF spaces
Weeks 5-6	Metencephalon (future pons/cerebellum) and myelencephalon (future medulla) differentiate	Sets the stage for cerebellar and cisternal development
Weeks 7-8	Rhombic lips form; cerebellar hemispheres begin to fuse at midline	Incomplete fusion may contribute to vermian hypoplasia (excluded in MCM)
Weeks 10–12	The foramen of Magendie (median aperture) opens, connecting the 4th ventricle to the cisterna magna	Critical for MCM: Delayed/absent opening may cause CSF accumulation
Weeks 12–20	Cerebellar vermis fully forms; cisterna magna matures	MCM is diagnosed if cisterna magna > 10 mm with normal vermis

**Table 2:** Differential diagnosis of posterior fossa anomalies.

Feature	Mega cisterna magna (MCM)	Blake's pouch cyst (BPC)	Dandy–Walker malformation (DWM)	Dandy–Walker variant (DWV)
Definition	Benign enlargement (> 10 mm) of the cisterna magna with normal cerebellum/vermis	Persistent Blake's pouch (embryonic CSF outlet) causing cystic dilation	Classic triad: 1. Vermis hypoplasia/agenesis 2. Cystic 4th ventricle dilation 3. Elevated tentorium	Partial vermian hypoplasia + mild 4th ventricle enlargement (less severe than DWM)

Key imaging findings	- Normal vermis and cerebellum - No mass effect - CSF-intensity space > 10 mm on MRI	- Cyst communicates with the 4th ventricle - Vermis may be elevated but intact - No cerebellar hypoplasia	- Absent/severely hypoplastic vermis - Large posterior fossa cyst - Tentorium elevated	- Partial vermian hypoplasia - Mild 4th ventricle enlargement - No tentorial elevation

Communication with the 4th ventricle	Yes (normal CSF flow)	Yes (cyst is a persistent Blake's pouch)	Yes (cyst replaces 4th ventricle)	Variable (often partial)

Cerebellar involvement	None	None (vermis may be compressed)	Severe hypoplasia/agenesis	Mild hypoplasia (inferior vermis)

Clinical implications	Typically asymptomatic; excellent prognosis if isolated	Often benign; may cause hydrocephalus if CSF flow is obstructed	High risk of neurodevelopmental delays, hydrocephalus (needs surgical intervention)	Variable; may have mild delays or be asymptomatic

Prenatal counseling	Reassurance if isolated	Monitor for hydrocephalus; usually favorable outcome	Poor prognosis; genetic testing recommended	Guarded prognosis; assess for associated anomalies

**Table 3:** Management recommendations for mega cisterna magna.

Clinical scenario	Action	Follow-up
Prenatal diagnosis	Confirm with fetal MRI if ultrasound shows cisterna magna > 10 mm. Exclude associated anomalies (e.g., vermian hypoplasia, ventriculomegaly)	If isolated: No further imaging unless new symptoms. Counsel parents on a benign prognosis
Isolated MCM (no symptoms)	No intervention needed. Reassure parents/patients	Clinical monitoring (e.g., annual pediatric neurodevelopmental exams until age 3)
MCM + other anomalies	Genetic testing (e.g., karyotype, microarray). Consult neurology/genetics	Serial imaging (MRI every 6–12 months) and multidisciplinary care (e.g., physical therapy/occupational therapy for delays)
Symptomatic MCM (e.g., headaches, hydrocephalus)	Re-evaluate imaging to rule out misdiagnosis (e.g., arachnoid cyst). Consult a neurosurgeon if there is obstructive hydrocephalus	Postsurgical monitoring is required if shunting/fenestration is performed
Postnatal incidental finding	MRI if prenatal studies were inconclusive or suboptimal	None if MRI confirms isolated MCM and the patient is asymptomatic

## Data Availability

No datasets were generated or analyzed during the current study.
